# Adults with possible food protein-induced enterocolitis syndrome with crustacean ingestion

**DOI:** 10.1186/s13223-020-00497-z

**Published:** 2020-11-11

**Authors:** Daniel H. Li, Andrew Wong-Pack, Andrea Leilani Macikunas, Harold Kim

**Affiliations:** 1 Department of Medicine, University of Toronto, Toronto, ON Canada; 2 Division of Clinical Immunology and Allergy, Department of Medicine, Western University, London, ON Canada; 3 Division of Clinical Immunology and Allergy, Department of Medicine, McMaster University, Hamilton, ON Canada

**Keywords:** Adult, Food protein-induced enterocolitis, Case study, Crustacean

## Abstract

**Background:**

Food protein-induced enterocolitis (FPIES), an entity previously thought to only affect children, has been increasingly described in adults. In this study, we report a Canadian cohort of 19 adolescents and adults with recurrent non-immunoglobulin E (IgE)-mediated gastrointestinal symptoms after crustacean ingestion, consistent with FPIES.

**Methods:**

We conducted a retrospective chart review of patients in an outpatient allergy clinic from January 2005 to May 2020. Electronic records were searched using keywords for crustaceans and for symptoms consistent with FPIES. We included patients with gastrointestinal symptoms specifically to crustaceans on more than one occasion, who were 14 years or older at the time of index reaction. Exclusion criteria included symptoms suggestive of an IgE-mediated anaphylactic reaction or a likely alternative diagnosis. We identified 19 patients for our cohort who met the criteria.

**Results:**

Our cohort was 68.4% female (13) and 32.6% (6) male. The average age at first reaction to crustaceans was 34 years old with a range of 14–68 years (median = 28 years; IQR = 32 years). Time from ingestion to beginning of symptoms ranged from 3 min to 6.5 h, with an average of 2.8 h (median = 2 h; IQR = 3.25 h). Duration of reaction ranged from less than a minute to over 48 h, with a mean of 9.4 h (median = 4 h; IQR = 7.75 h). Patients had 4.8 reactions on average; however, number of reactions ranged from 2 to 12.5 (median = 3, IQR = 3). All patients identified a “trigger” food in the crustacean group, and 12 subjects identified additional reactions to other seafood.

**Conclusions:**

This case series will better characterize and advance our understanding of this disease entity in adults. There are key differences in the presentation of FPIES in adults compared to children, namely female predominance, difference in solid food trigger, and unpredictable time course. Future studies are needed to examine the pathophysiology and natural history of adult FPIES. Specific guidelines should be developed for the diagnosis and management in adults.

*Trial registration:* retrospectively registered.

## Background

Food protein-induced enterocolitis syndrome (FPIES) is a non-immunoglobulin E (IgE)-mediated gastrointestinal food hypersensitivity that was previously thought to only affect infants and young children. It typically affects the entire gastrointestinal tract and manifests with symptoms of profuse emesis and diarrhea that may lead to dehydration and lethargy in the acute setting [1]. In the chronic setting, watery diarrhea with intermittent vomiting may lead to weight loss, failure to thrive, and metabolic derangements [2, 3]. In the majority of children, FPIES is caused by a single food with the most common implicated foods being cow’s milk and soy [4].

There is now emerging research describing a similar non-IgE mediated food allergy in adult patients [5]. It appears from the current literature that shellfish (crustaceans and molluscs) are the most common food described to cause symptoms consistent with FPIES in adults [6–8]. In accordance with the FPIES guideline, the diagnosis of FPIES is made if there is evidence of a supportive medical history and resolution of symptoms with elimination of the causative food, or oral food challenge confirmation if the history is unclear [9]. In this study, we report a Canadian cohort of 19 adults and adolescents with recurrent non-IgE mediated gastrointestinal symptoms consistent with FPIES to crustaceans.

## Methods

We conducted a retrospective chart review of patients assessed in a referral only outpatient allergy clinic for possible crustacean allergy from January 2005 to May 2020. Electronic records were searched using keywords for crustaceans: “shrimp, lobster, crab, shellfish”, and for symptoms possibly consistent with FPIES: “vomiting, diarrhea, abdominal pain”. We included patients with more than one instance of gastrointestinal symptoms specifically to crustaceans, who were 14 years or older at the time of index reaction. Exclusion criteria included symptoms suggestive of an IgE-mediated anaphylactic reaction or a likely alternative diagnosis such as inflammatory bowel disease, lactose intolerance, or celiac disease. The study met approval from the Hamilton Integrated Research Ethics Board (HiReb) of McMaster University.

One hundred and ninety-one patients met initial search query for possible adult FPIES. After exclusion of patients under 14 years of age or history suggestive of an alternative diagnosis, 19 patients were included in our cohort (Table 1).

**Table 1:** Demographics of patients with FPIES-like symptoms after ingestion of crustaceans (n = 19)

Characteristic	n	% or IQR
Sex		
Female	13	68.4
Male	6	31.6
Age at onset of symptoms	34.3	20.5–52.5
Delay to presentation (years)	13.4	4.0–13.5
Type of crustacean reacted to*		
Shrimp	18	94.7
Lobster	6	31.6
Crab	5	26.3
Symptoms		
Emesis	19	100
Abdominal pain	7	36.8
Diarrhea	8	42.1
Length of reaction (hours)	9.4	2.0–9.75
Time after ingestion (hours)	2.9	1.5–4.75
Number of reactions	4.8	3–6
Positive skin test		
Dust mite	6	31.6
Aeroallergen (other)	5	26.3
Atopy	3	15.8
Emergency Department		
Yes	3	15.8
No	16	84.2

## Results

**Fig. 1:**
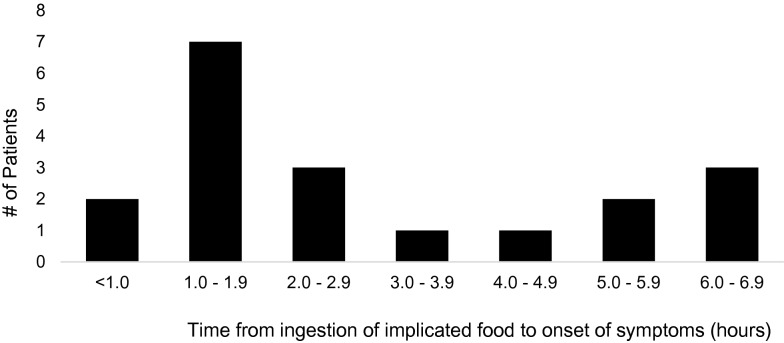
Time from ingestion to onset of symptoms in adult patients with FPIES-like symptoms, measured in hours

Our cohort was 68.4% female (13) and 32.6% (6) male. The average age at first reaction to crustaceans was 34 years old with a range of 14–68 years (median = 28 years; IQR = 32 years). Time from ingestion to beginning of symptoms (Fig. 1) ranged from 3 min to 6.5 h, with an average of 2.8 h (median = 2 h; IQR = 3.25 h). Duration of reaction ranged from 1 min to over 48 h, with a mean of 9.4 h (median = 4 h; IQR = 7.75 h). All patients reported vomiting, while 42.1% of patients reported diarrhea and 36.8% reported abdominal pain. Patients had 4.8 reactions on average; however, number of reactions ranged from 2 to 12.5 (median = 3, IQR = 3). All patients identified a food in the crustacean group as the implicated food for their reaction. Twelve subjects identified reactions to other seafood in addition to their original reaction with crustaceans. Three patients sought care at the Emergency Department for their reactions; none were hospitalized (Additional file 1: Figure 1 and Table 1).

Five patients, of 18 who were skin prick tested, had a positive result (27.8%) to 1 of the common aeroallergens. Of these, 17 patients underwent skin prick testing specifically for dust mite (*Dermatophagoides pteronyssinus* and *Dermatophagoides farinae)* and 6 patients were positive (35.3%). None had personal history or family history of FPIES, although 3 patients had a history of atopy (allergic rhinitis, asthma, atopic dermatitis, food allergy).

## Discussion

FPIES is a heterogeneous, non-IgE-mediated gastrointestinal food hypersensitivity that has been anecdotally reported in adults for many years, but only recently have there been case series published in the peer-reviewed literature describing this clinical entity in adult patients [5–8, 10]. We hope our case series will better characterize and advance our understanding of this disease entity in adults. In our study, a Canadian cohort of 19 adolescent and adult patients presented with recurrent gastrointestinal symptoms after ingestion of a crustacean. The interval between ingestion of the implicated food and the onset of symptoms (mean of 2.8 h) and reproducible symptoms to the food (4.8 reactions on average) is typical for a non-IgE mediated reaction. None of our patients had extra-gastrointestinal manifestations that later progressed to anaphylactic reactions.

We know from the literature that up to 25 percent of children and infants fulfilling the diagnostic criteria for FPIES have or develop IgE antibodies to the trigger food, possibly due to avoidance or pre-existing food allergy [4, 11]. One of our patients was skin-prick test positive to shrimp as well as to *D. farinae*, which we attributed to tropomyosin cross-reactivity. Clinically, her reaction was consistent with a non-IgE reaction given her gastrointestinal predominant symptoms and the time course of her reaction. As such, having a positive skin prick test does not preclude the diagnosis of FPIES and we chose to include her in the cohort.

There are some key differences in the clinical features of FPIES in adults when compared to children. We noticed a predominance of female patients (68.4%) with adult FPIES, as opposed to a male predominance of 50%-60% observed in children [4]. This is consistent with Du et al. and Gonzalez-Delgado et al. who observed a 90% and 88% female predominance in their cohorts of adult FPIES patients respectively [5, 8]. This may suggest a hormonal role in the pathogenesis of FPIES. Our findings of shrimp as the predominate food trigger for adult FPIES is consistent with previous cases studies in adult patients [5, 8]. This is in contrast to cow’s milk and soy as the most common food trigger in FPIES in children [4]. We did not find a strong correlation between a history of atopy and FPIES in adults (3 out of 19 patients), which is something often noted in children. This contrasts with Gonzalez-Delgado et al. who noted 72% of their adult and adolescent patients with FPIES had an atopic background [8]. Our cohort reported a large range of time to onset of symptoms after ingestion of food (range of 3 min to 6.5 h), instead of the typical 1 to 4 h suggested for pediatric FPIES. This may suggest that the time course of adult FPIES is less predictable than pediatric FPIES and may occur more rapidly [9]. In children, solid food triggers for FPIES tend to vary based on geographic location as noted in previous studies involving children in Australia and Spain [12, 13]. Interestingly, despite differences in geographic location in our Canadian cohort of patients, we saw predominately crustaceans as the implicated food in adult FPIES. This was similar to the findings of Gonzalez-Delgado et al., who conducted their study in Spain [8]. Given the differences between adult FPIES and classical FPIES in the pediatric population, specific guidelines should be developed for diagnosis and management of FPIES in adults.

Little is known concerning the pathogenesis of FPIES. It is hypothesized that the ingestion of food allergens causes a T-cell mediated response that results in local inflammation leading to increased intestinal permeability and fluid shift [14]. There may also be a role for transforming growth factor beta (TGF-β) receptors, activated peripheral blood monocular cells, and increased tumor necrosis factor (TNF-α) alpha in the intestinal mucosa involved in intestinal inflammation [15]. Ondansetron has been shown to be beneficial in the treatment of FPIES in children, though the mechanism is unclear [16]. To date, there have not been studies on its use in adult patients with FPIES; further research should be conducted to determine if it is effective in adults as well.

Adult FPIES is a newly recognized disease entity and the natural history of this condition is not well known. As such, there is often a significant delay in the time of symptom onset to diagnosis. We noted a prolonged delay in diagnosis with a median of 10 years from symptom onset to diagnosis, comparable to a median of 8 years noted by Gonzalez-Delgado et al. [8]. To aid in decreasing the delay in diagnosis of FPIES in adults, it is important to educate other medical specialties including gastroenterology, emergency medicine, and family medicine concerning this disease entity. It is also important to increase awareness of FPIES in adult patients, given the possibility of a severe reaction which may result in serious dehydration and volume depletion.

Limitations to our study include potential recall bias given its retrospective nature, as well as lack of confirmatory testing. Not all patients had serum specific IgE-testing or serum tryptase drawn at time of reaction. Not all patients had oral food challenges completed as many were not willing to undergo the challenge and preferred strict avoidance of the implicated food.

## Conclusion

Currently, there is no definite guideline defining an FPIES-like syndrome in adults. Given that adult onset shrimp allergy is a well described condition, we recommend patients who experience a reaction to shrimp or other crustaceans be prescribed an epinephrine autoinjector and recommend strict avoidance until evaluation by an allergy and immunology specialist. Other specialists, including gastroenterologists, emergency physicians, and family medicine practitioners should be aware of FPIES when evaluating adults with reactions to shellfish. Patients acutely presenting with intractable vomiting or signs and symptoms of hypovolemia should present to the Emergency Department for evaluation and potential fluid resuscitation. Ondansetron may be considered for symptom management given its use in the treatment of paediatric patients, but further studies are needed in the adult population [16]. Future studies examining the pathophysiology of adult FPIES and its natural history are needed. In addition, observed trends such as the association with crustaceans in the adult population and a female predominance in the condition should be explored in the future [5, 8].

## Supplementary information

**Additional file 1: Figure 1.** Foods associated with FPIES-like reaction in adult patients. **Table 1.** Description of patients with FPIES-like reactions to crustaceans.

## Data Availability

All data generated or analysed during this study are included in this published article [and its Additional information files].

## Abbreviations

FPIES: Food protein-induced enterocolitis syndrome
IgE: Immunoglobulin E
HiReb: Hamilton Integrated Research Ethics Board
IQR: Interquartile range
TGF-β: Transforming growth factor beta
TNF-α: Tumor necrosis factor alpha
