# Supplementary material for: Adults with possible food protein-induced enterocolitis syndrome with crustacean ingestion
Source: Allergy Asthma Clin Immunol. 2020 Nov 11;16:99. doi: 10.1186/s13223-020-00497-z (PMC7659059; doi:10.1186/s13223-020-00497-z)
Supplement: Supplementary file 1 — Additional file 1: Figure 1. Foods associated with FPIES-like reaction in adult patients. Table 1. Description of patients with FPIES-like reactions to crustaceans. [file 13223_2020_497_MOESM1_ESM.docx]

**Additional Figure 1: Foods Associated with FPIES-like Reaction in Adult Patients**

Foods associated with FPIES-like reaction in cohort of adult patients. N = 19; some patients reacted to more than one food.

|  | **Sex** | **Food** | **Age at Onset (years)** | **Time after eating (hours)** | **Length of reaction (hours)** | **Assessment Delay (years)** | **Emesis** | **Abdo Pain** | **Diarrhea** | **ER Visit** | **OC Crust.** | **SPT Crust.** | **SPT DM** | **SPT Aero** | **sIgE Crust.** |
| --- | --- | --- | --- | --- | --- | --- | --- | --- | --- | --- | --- | --- | --- | --- | --- |
| 1 | F | shrimp | 15 | 2 to 3 | 48 | 7 | + | + | + | + | n.d. | - | - | - | n.d. |
| 2 | F | shrimp, lobster | 20 | 1 to 3 | 3 to 7 | 14 | + |  |  |  | n.d. | - | - | - | - |
| 3 | F | shrimp, crab, clam | 29 | 5 | 2 to 3 | 2 | + |  |  |  | n.d. | - | - | - | n.d. |
| 4 | F | shrimp | 50-55 | 1.5 | 2 to 3 | 13.5 | + |  | + | + | Shrimp: pos | - | - | - | n.d. |
| 5 | M | shrimp | 25-26 | 2 | 6 to 8 | 3.5 | + |  |  |  | n.d. | - | - | - | n.d. |
| 6 | F | lobster, crab, scallop | 18 | 3 | 3 to 4 | 7 | + |  |  |  | n.d. | - | n.d | + | n.d. |
| 7 | F | shrimp | 68 | 2 to 11 | 8 to 12 | 3 | + |  |  |  | n.d. | - | + | + | - |
| 8 | M | shrimp | 25 | 3 to 6 | > 48 | 56 | + |  |  |  | n.d. | - | - | + | n.d. |
| 9 | M | shrimp lobster | 14 | 1.5 | 12 to 14 | 44 | + | + | + |  | n.d. | - | border | - | n.d. |
| 10 | M | shrimp, lobster | 33 | 0.05 | 1 | 10 | + |  |  |  | n.d. | - | + | - | n.d. |
| 11 | F | shrimp, mussels, scallop | 32 | 0.5 to 1 | 0.01 | 4 | + |  |  |  | n.d. | - | - | - | n.d. |
| 12 | M | shrimp, tuna | 18-20 | 0.5 to 3 | 0.5 to 1.5 | 10 | + | + | + |  | n.d. | - | + | - | n.d. |
| 13 | F | shrimp, crab | 55 | 1.5 | 1 to 2 | 11 | + |  |  |  | n.d. | - | - | - | n.d. |
| 14 | F | clam, shrimp, tuna, fish, scallop, mussel | 50-55 | 1 to 2 | 12 | 13.5 | + | + | + | + | Clam: neg | + | + | - | + |
| 15 | F | shrimp | 22 | 6 to 7 | 2 to 3 | 4 | + | + | + |  | n.d. | - | + | + | n.d. |
| 16 | F | mussels, crab, shrimp | 21 | 6 | 1 | 4 | + |  |  |  | n.d. | - | - | - | n.d. |
| 17 | M | shrimp | 55 | 1 to 1.5 | 6 | 8 | + | + | + |  | n.d. | - | n.d | n.d | n.d. |
| 18 | F | scallops, shrimp, clam, lobster | 67 | 5 | 7 to 12 | 10 | + |  | + |  | n.d. | - | - | - | n.d. |
| 19 | F | shrimp, crab, lobster | 28 | 1 to 2 | 3 to 5 | 30 | + | + |  |  | Shrimp: neg, Scallop: neg | - | + | + | - |

‘’

**Additional Table 1: Description of Patients with FPIES-like Reactions to Crustaceans**

Patients included in study numbered 1 to 19 with demographic data, clinical, and diagnostic data
*Abbreviations: OC = Oral challenge, Crust = crustacean which was implicated in reaction; SPT = skin-prick test, DM = dust mite, Aero = aeroallergen, sIgE Crust = serum specific IgE measurement, n.d. = test not done*
